# Author Correction: *Atrx* inactivation drives disease-defining phenotypes in glioma cells of origin through global epigenomic remodeling

**DOI:** 10.1038/s41467-021-27820-5

**Published:** 2022-01-05

**Authors:** Carla Danussi, Promita Bose, Prasanna T. Parthasarathy, Pedro C. Silberman, John S. Van Arnam, Mark Vitucci, Oliver Y. Tang, Adriana Heguy, Yuxiang Wang, Timothy A. Chan, Gregory J. Riggins, Erik P. Sulman, Frederick F. Lang, Chad J. Creighton, Benjamin Deneen, C. Ryan Miller, David J. Picketts, Kasthuri Kannan, Jason T. Huse

**Affiliations:** 1 Department of Translational Molecular Pathology, University of Texas MD Anderson Cancer Center, Houston, TX 77030 USA; 2 Human Oncology and Pathogenesis Program, Memorial Sloan-Kettering Cancer Center, New York, NY 10065 USA; 3 Department of Pathology, University of Texas MD Anderson Cancer Center, Houston, TX 77030 USA; 4 Department of Pathology and Laboratory Medicine, University of North Carolina School of Medicine, Chapel Hill, NC 27516 USA; 5 Department of Pathology, New York University School of Medicine, New York, NY 10016 USA; 6 Department of Radiation Oncology, Memorial Sloan-Kettering Cancer Center, New York, NY 10065 USA; 7 Departments of Neurosurgery, Oncology, and Genetic Medicine, Johns Hopkins School of Medicine, Baltimore, MD 21231 USA; 8 Department of Radiation Oncology, University of Texas MD Anderson Cancer Center, Houston, TX 77030 USA; 9 Department of Neurosurgery, University of Texas MD Anderson Cancer Center, Houston, TX 77030 USA; 10 Department of Medicine and Dan L. Duncan Comprehensive Cancer Center Division of Biostatistics, Baylor College of Medicine, Houston, TX 77030 USA; 11 Department of Neuroscience, Baylor College of Medicine, Houston, TX 77030 USA; 12 Departments of Pharmacology and Neurology, Lineberger Comprehensive Cancer Center and Neuroscience Center, University of North Carolina School of Medicine, Chapel Hill, NC 27516 USA; 13 Department of Biochemistry, Microbiology, and Immunology, University of Ottawa, Ottawa, ON K1H 8L6 Canada; 14 Ottawa Hospital Research Institute, Ottawa, ON K1H 8L6 Canada

Correction to: *Nature Communications* 10.1038/s41467-018-03476-6, published online 13 March 2018.

The original version of this article contained an error in the author list. The author, Frederick F. Lang, was incorrectly listed as Frederick Lang. This error has been corrected in the HTML and PDF versions of the article.

