# Supplementary material for: Atrx inactivation drives disease-defining phenotypes in glioma cells of origin through global epigenomic remodeling
Source: Nat Commun. 2018 Mar 13;9:1057. doi: 10.1038/s41467-018-03476-6 (PMC5849741; doi:10.1038/s41467-018-03476-6)
Supplement: Supplementary file 1 — Supplementary Information [file 41467_2018_3476_MOESM1_ESM.pdf]

Atrx inactivation drives disease-defining phenotypes in glioma cells of origin through global epigenomic remodeling

Danussi C et al.

Supplementary Information

Contents

Supplementary Tables: pp. 2-3

Supplementary Figures: pp. 4-14

gene	p-value	fold change	chr	trans start	trans stop	ATAC Closed	ATAC Open	Atrx ChIP
APOE	0.000233264	7.62E+159	chr7	20273596	20293596	0	8072.37	1.75E+07
Rnd2	9.49E-05	1.11E+16	chr11	101316312	101336312	0	13662.8	3.36E+06
Mfge8	1.92E-05	4.04E+15	chr7	86283946	86303946	0	3574.48	703953
Agt	3.19E-05	2.70E+12	chr8	127083606	127103606	0	2902.71	2.94E+06
TRAF4	0.000440314	1.30E+07	chr11	77969018	77989018	0	6661.14	1.40E+07
Fgfr3	0.000193534	856082	chr5	34054372	34074372	0	3563.29	1.94E+06
Carhsp1	8.79E-07	197904	chr16	8662246	8682246	0	6206.48	1.96E+06
DDR1	0.00839422	4871.93	chr17	35827497	35847497	2073.71	9895.72	2.25E+07
Zfp703	0.000155961	1948.75	chr8	28077807	28097807	16190.8	8558.01	1.05E+07
Tyro3	9.15E-06	1878.75	chr2	119613468	119633468	0	6374.52	3.75E+06
Plxnb3	1.70E-05	1275	chrX	70992428	71012428	0	9026.8	1.87E+06
PTGDS	0.000881781	880.492	chr2	25314677	25334677	0	13478.1	2.60E+06
Vat1	0.000655119	859.256	chr11	101317544	101337544	0	8831.87	946019
Nkx6-2	3.33E-06	393.774	chr7	146758689	146778689	4642.27	2529.03	178753
Emp1	6.26E-05	262.731	chr6	135302948	135322948	0	6992.24	5.70E+06
CDK5	0.000150548	244.195	chr5	23919348	23939348	0	1928.62	836143
Hspa2	0.00100924	151.225	chr12	77495162	77515162	0	5348.84	2.96E+06
Emilin1	0.000135772	148.816	chr5	31205774	31225774	0	10762.3	1.80E+06
Cdkn1c	6.13E-06	114.475	chr7	150636955	150656955	0	9622.99	142871
TMEM176B	0.0058633	84.2633	chr6	48778305	48798305	0	11953.2	2.91E+06
GNA13	0.000226585	31.602	chr11	109214144	109234144	2845.12	3874.08	2.25E+07
NR1D1	5.28E-05	31.3837	chr11	98621740	98641740	0	2061.39	2.26E+06
Dpysl4	0.000293476	24.782	chr7	146261899	146281899	0	5721.1	646879
Rap1gap	0.000380142	24.1676	chr4	137210640	137230640	1232.62	1951.07	2.87E+06
Cyr61	4.22E-05	21.9173	chr3	145302949	145322949	0	3846.3	1.15E+07
ATG12	0.000383878	20.4283	chr18	46891233	46911233	0	3369.51	2.35E+06
RAC1	0.00590689	18.1129	chr5	144268476	144288476	0	2995.2	1.85E+06
Zmiz2	5.86E-05	16.657	chr11	6279076	6299076	0	6549.82	125718
STAT3	0.000514096	13.6311	chr11	100741626	100761626	0	9028.35	7.16E+06
Nkx2-1	4.38E-06	10.2444	chr12	57627895	57647895	0	2059.05	112714
Dpysl3	0.000100348	9.59497	chr18	43543031	43563031	387.162	918.255	645196
Plin3	3.16E-05	8.46429	chr17	56419934	56439934	0	8731.07	1.39E+06
PIK3R1	0.000423348	8.08984	chr13	102452602	102472602	1442.9	1645.38	1.05E+07
Pdgfra	0.00011443	6.74092	chr5	75538315	75558315	0	10274.2	6.99E+06
NTRK2	6.97E-05	6.18145	chr13	58897956	58917956	0	3229.01	1.68E+07
NOTCH1	0.00557255	5.35315	chr2	26308267	26328267	0	3336.58	986567
Tspan4	0.000463638	4.92382	chr7	148651138	148671138	7301.73	10317	631092
TIMP2	0.000412584	2.27031	chr11	118206820	118226820	0	3410.04	2.52E+06

Supplementary Table 1: Genes selected for siRNA knockdown in this study. Fold change between Atrx- and Atrx+ mNPCs is shown along with associated *P* value. Associations with Atrx ChIP-seq peaks and ATAC-seq open and closed regions are determined by summing enrichment peak area within a ± 10 kb window surrounding the transcriptional start site in question. Transcriptional start and stop sites are also shown.

Antibody	Application	Company	Catalog Number	Concentration
Atrx (H-300)	ChIP; WB	Santa Cruz	sc-15408	40 ug; 1 : 200
H3.3	ChIP	Millipore	09-838	20 ug
p53 (1C12)	WB	Cell Signaling Technology	2524	1 : 1000
Vinculin	WB	Sigma Aldrich	V9264	1 : 5000
RhoA	WB	Cytoskeleton Inc.	ARH04	1 : 200
GFAP	WB	Abcam	ab7260	1 : 10.000
Tubb3	WB; IF	Abcam	ab7751	1 : 1000
Olig2	WB	Millipore	AB9610	1 : 2500
IDH R132H	WB	Dianova	DIA-H09	1 : 500
GNA13	WB; IHC	Abcam	ab128900	1 : 1000; 1 : 250
Caspase 3	WB	Cell Signaling Technology	9665	1 : 1000
Cleaved Caspase 3	WB	Cell Signaling Technology	9664	1 : 1000

Supplementary Table 2: Antibodies used in this study. Application, commercial source, catalog number, and concentration of use also given.

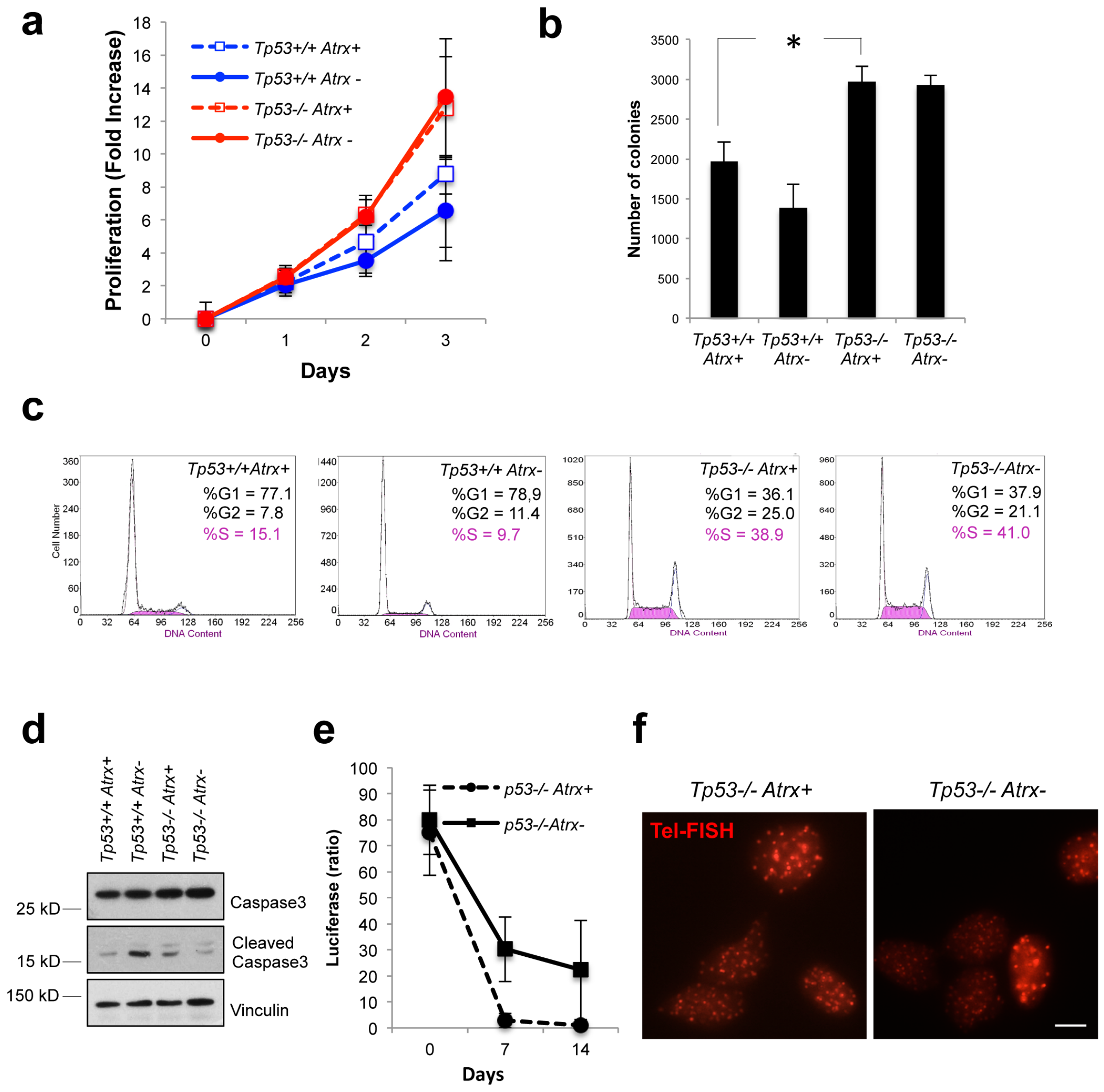

Supplementary FIG. 1: Functional consequences of Atrx deficiency in mNPCs. (a-b) Proliferation of mNPCs in adherent conditions (laminin coated plates, a) and in soft agar (b, *P = 0.002; unpaired two-tailed t-test). Plots represent the average of three biological replicates. Error bars reflect standard deviation. c, propidium iodide cell cycle FACS analysis of mNPCs. d, western blot showing elevated cleaved caspase 3 in *Tp53+/+ Atrx-* mNPCs (Vinculin loading control). e, luciferase quantification of *in vivo* growth of *Tp53-/- Atrx+* and *Tp53-/- Atrx-* mNPCs intracranially implanted into nude mice and monitored for 14 days; bars indicate standard error (9 mice/genotype). f, telomere FISH in *Tp53-/- Atrx+* and *Tp53-/- Atrx-* mNPCs showing no evidence of ALT in either line (scale bar 5 μm).

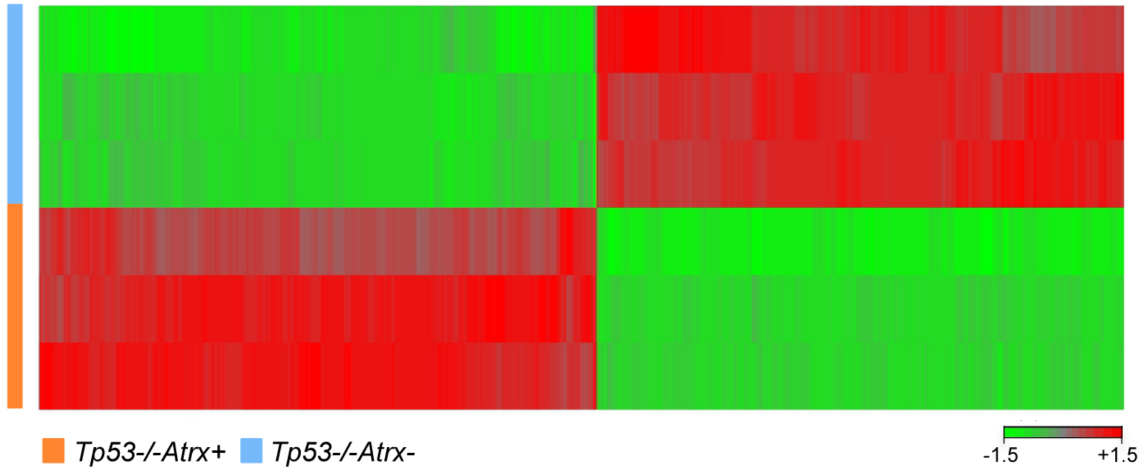

Supplementary FIG. 2: Differential gene expression in *Tp53*-/- mNPCs arising with Atrx deficiency. Heat map of differentially expressed genes (fold change > 2, $Q < 0.01$; ANOVA FDR test) in *Tp53*-/- *Atrx*+ and *Tp53*-/- *Atrx*- mNPCs.

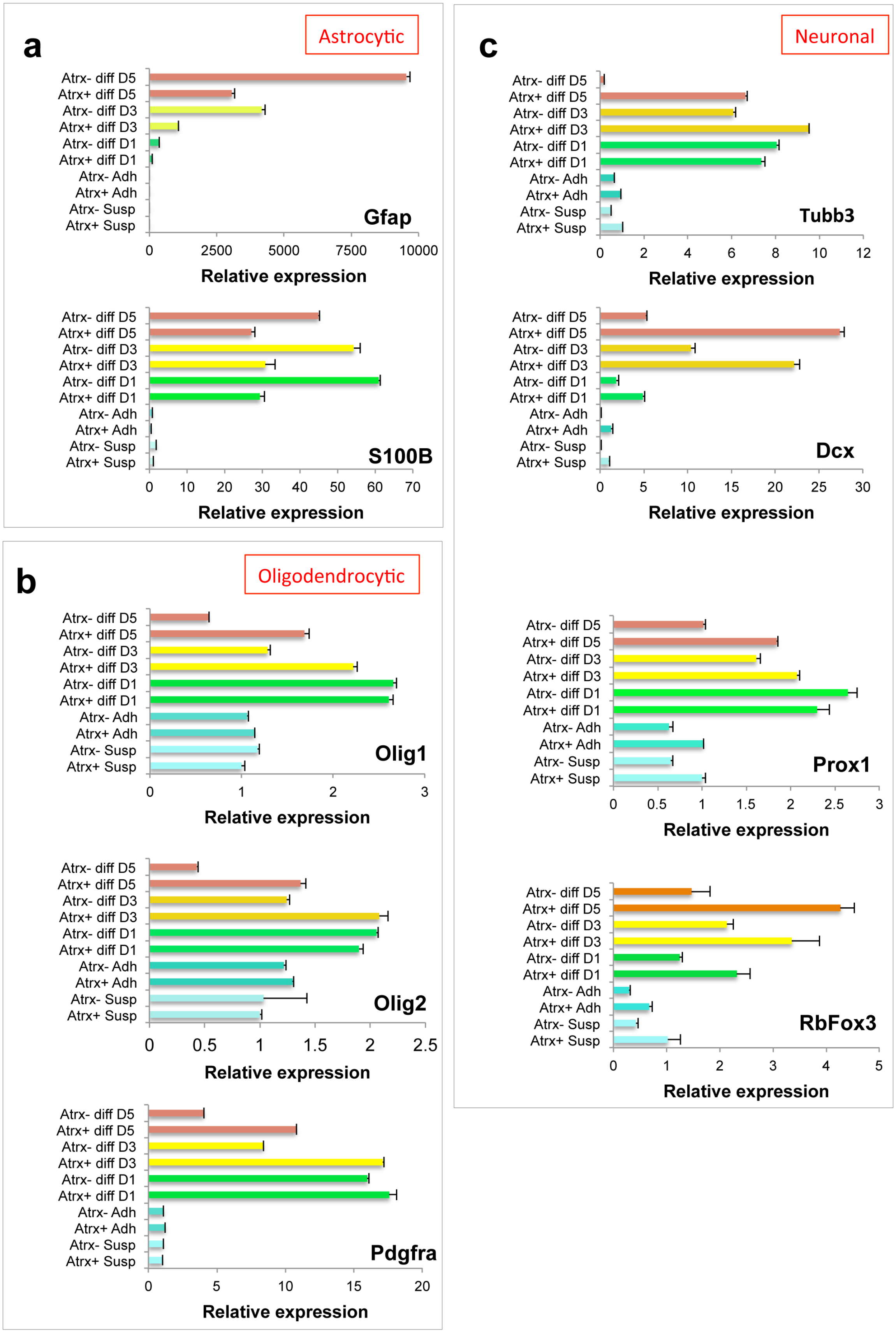

a
Astrocytic
Atrx- diff D5
Atrx+ diff D5
Atrx- diff D3
Atrx+ diff D3
Atrx- diff D1
Atrx+ diff D1
Atrx- Adh
Atrx+ Adh
Atrx- Susp
Atrx+ Susp
Gfap
0 2500 5000 7500 10000
Relative expression
S100B
0 10 20 30 40 50 60 70
Relative expression
b
Oligodendrocytic
Olig1
0 1 2 3
Relative expression
Olig2
0 0.5 1 1.5 2 2.5
Relative expression
Pdgfra
0 5 10 15 20
Relative expression
c
Neuronal
Tubb3
0 2 4 6 8 10 12
Relative expression
Dcx
0 5 10 15 20 25 30
Relative expression
Prox1
0 0.5 1 1.5 2 2.5 3
Relative expression
RbFox3
0 1 2 3 4 5
Relative expression

Supplementary FIG. 3: Atrx deficiency alters mNPC differentiation profiles. RT-qPCR analysis of astrocytic, oligodendrocytic and neuronal markers in *Atrx*+ and *Atrx*- mNPCs (3 replicates per genotype) cultured in suspension (Susp), on laminin (Adh) and in differentiation conditions for the indicated number of days (D1-D5). Error bars reflect standard deviation.

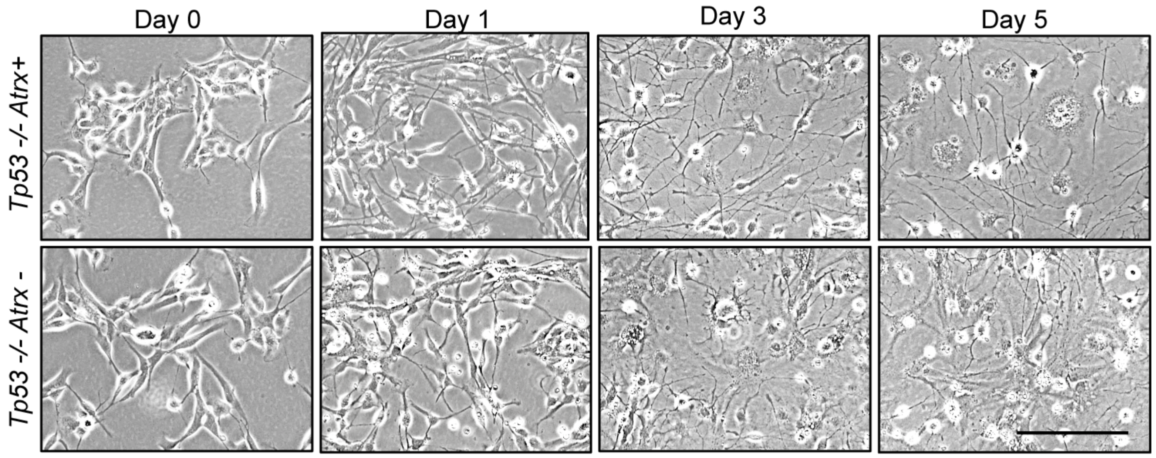

Supplementary FIG. 4: Atrx deficiency impairs neuronal differentiation in mNPCs. Phase-contrast micrographs of *Atrx*+ and *Atrx*- mNPCs cultured in differentiation conditions for 1, 3 and 5 days (scale bar 50 μm).

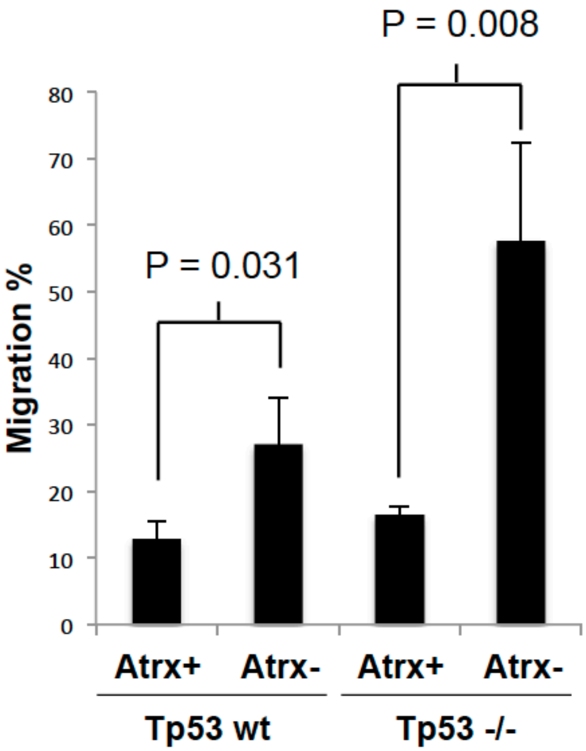

Supplementary FIG. 5: Atrx deficiency promotes mNPC motility. Transwell assay showing increased migratory behavior in *Atrx*- mNPCs, regardless of *Tp53* status. The impact of *Tp53* inactivation on mNPC motility was not significant (*P* = 0.098). Error bars reflect standard deviation. *P* values determined by unpaired two-tailed t-test.

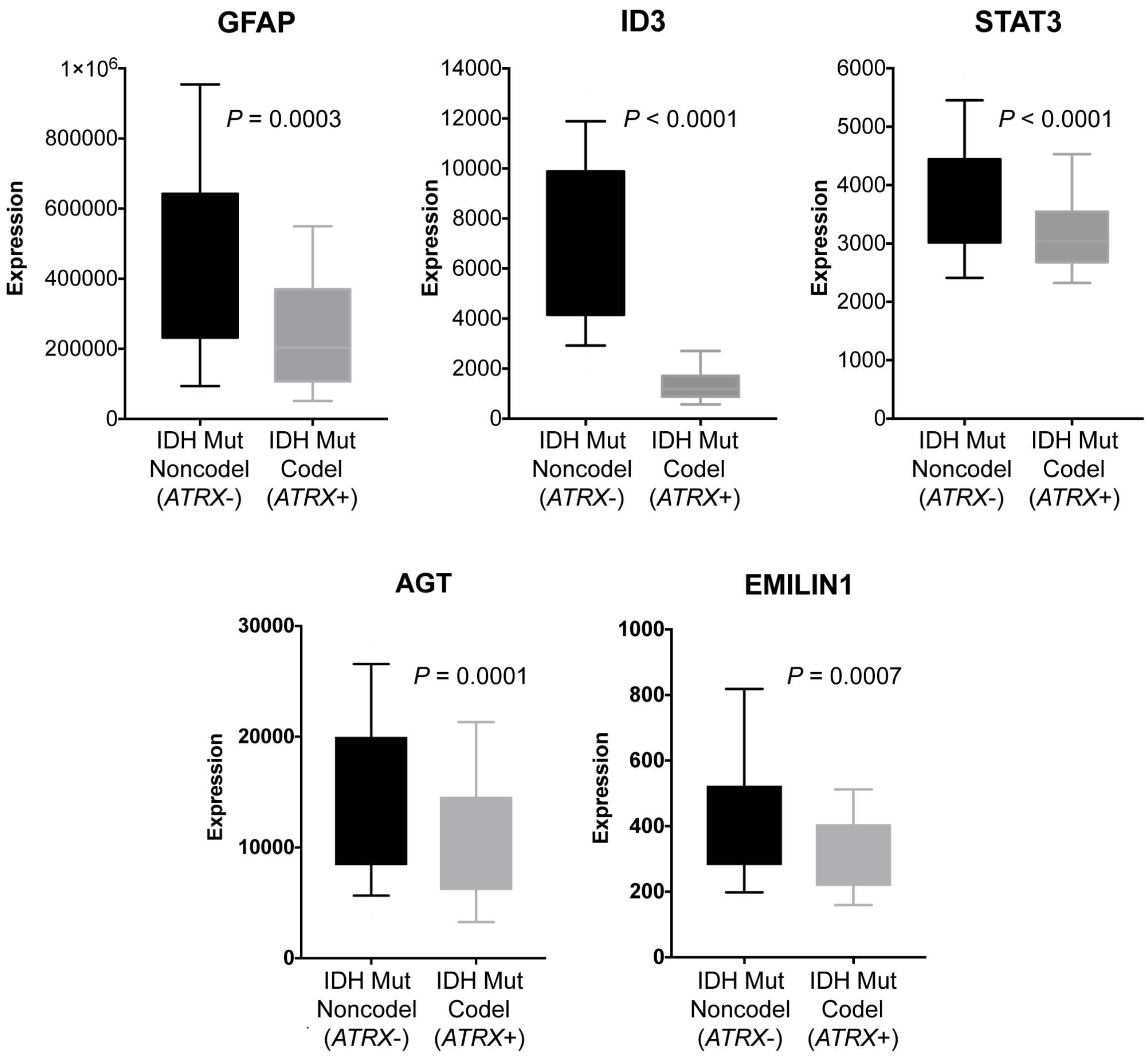

Supplementary FIG. 6: Astrocytic marker/regulator and cell motility effector expression is upregulated in *ATRX*- human gliomas. LGG expression data from TCGA showing levels of *GFAP, ID3, STAT3, AGT, and EMILIN1* expression in ATRX-intact (*ATRX*+) and ATRX-deficient (*ATRX*-) gliomas. Error bars reflect the 10-90 percentile spread. *P* values determined by unpaired two-tailed t-test.

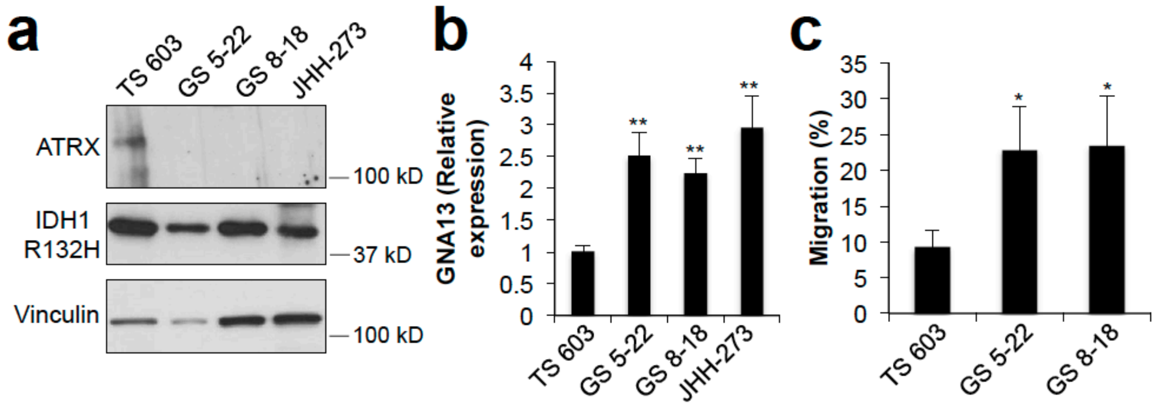

Supplementary FIG. 7: ATRX deficiency downregulates GNA13 and increases cellular motility in GSCs. a, western blot (a) showing IDH1 R132H and ATRX expression in TS 603, GS 5-22, GS 8-18, and JHH-273 GSCs (Vinculin loading control). JHH-273 cells are only passaged as xenografts. b, RT-qPCR (3 replicates) showing increased GNA13 expression across ATRX-deficient GSCs relative to TS 603. c, transwell migration assay (5 replicates) demonstrating increased motility in both GS 5-22 and GS 8-18 GSCs relative to TS 603 (*$P = 0.02$, **$P < 0.003$; unpaired two-tailed t-test; error bars reflect standard deviation).

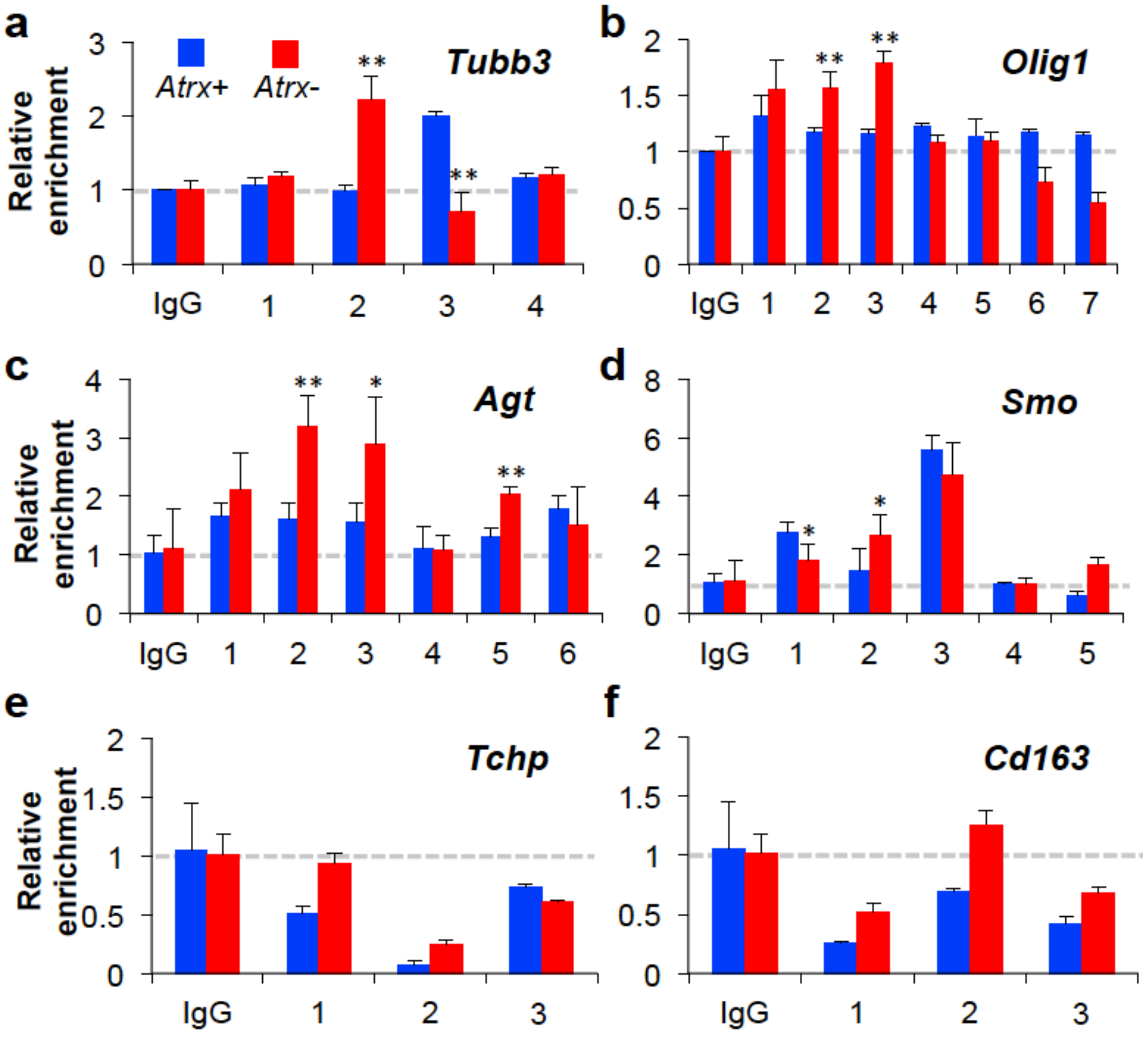

Supplementary FIG. 8: Alterations in H3.3 composition at Atrx binding sites associated with Atrx-dependent differential gene expression. H3.3 ChIP qPCR from Atrx-bound genomic loci approximating genes differentially expressed in the context of Atrx deficiency (a-d). In each case, multiple regions were amplified across the locus of interest (*$P < 0.05$, **$P < 0.005$; unpaired two-tailed t-test). H3.3 was not enriched over baseline (IgG) in association with two genes (*Tchp* and *Cd163*) not associated with Atrx-dependent differential expression (e-f). Error bars reflect standard deviation.

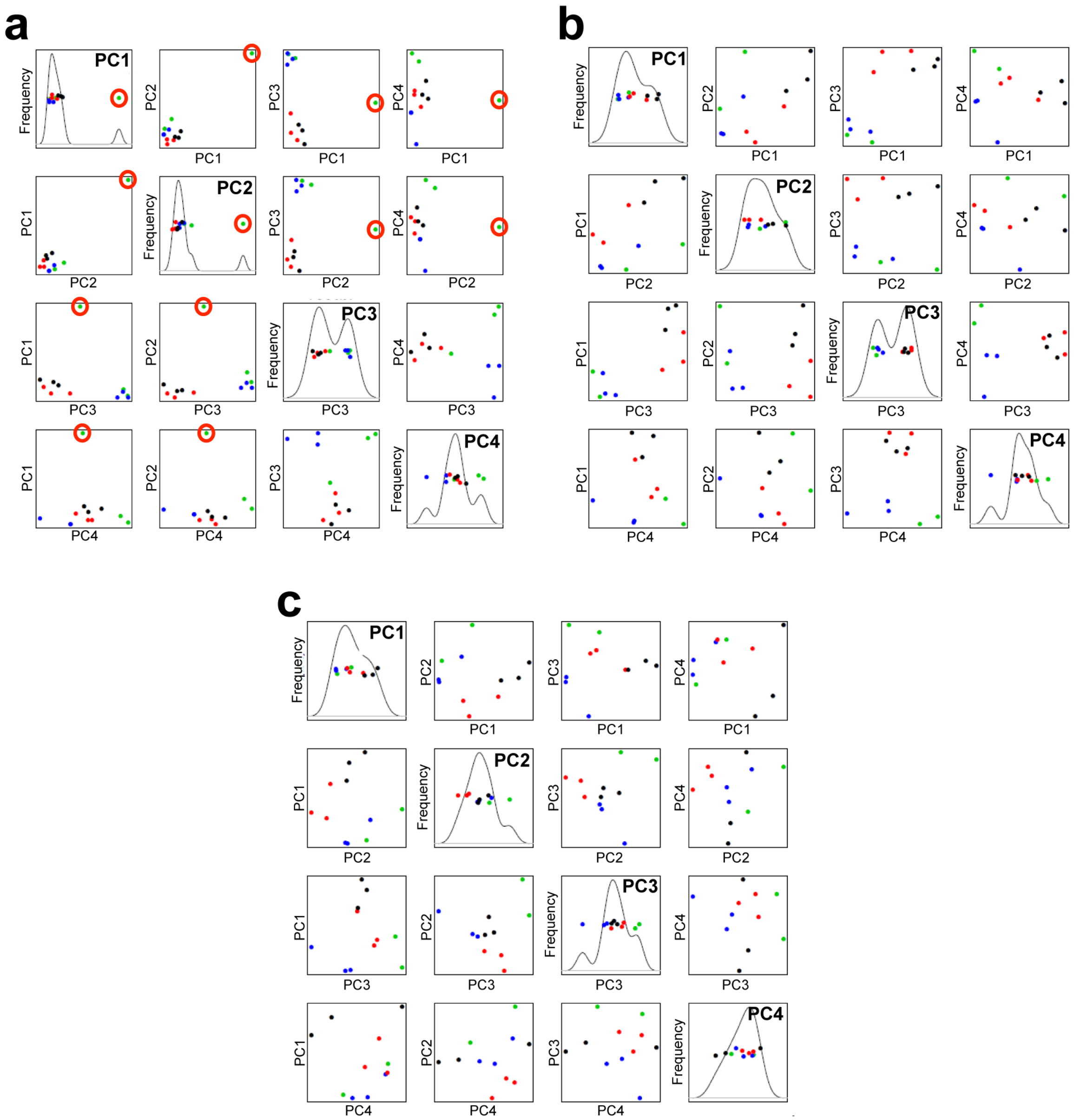

Supplementary FIG. 9: Principal component analysis (PCA) of mNPC RNA-seq data. PCA of raw data (a) reveals outlier sample (red circle) most prominently with reference to PC1 and PC2. b, PCA following exclusion of outlier sample. c, PCA following exclusion of outlier sample and median centering. mNPC genotypes indicated as follows: black: *Tp53*+/+ *Atrx*+, red: *Tp53*+/+ *Atrx*-, blue: *Tp53*-/- *Atrx*+, green: *Tp53*-/- *Atrx*-.

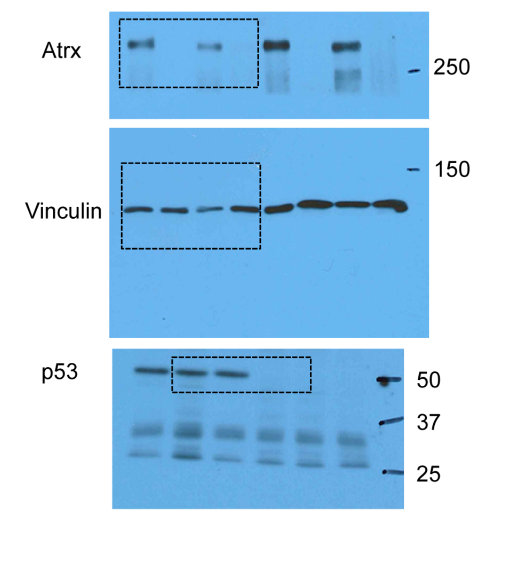

Figure 1a

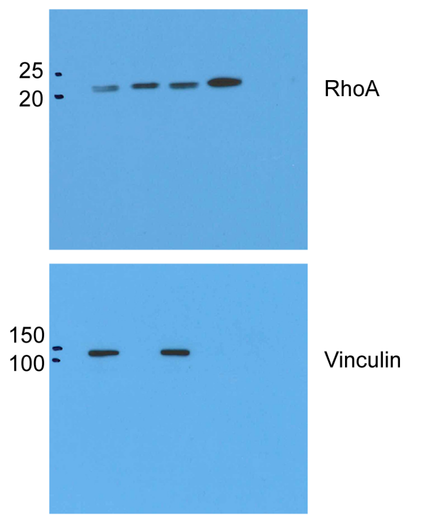

Figure 3 d

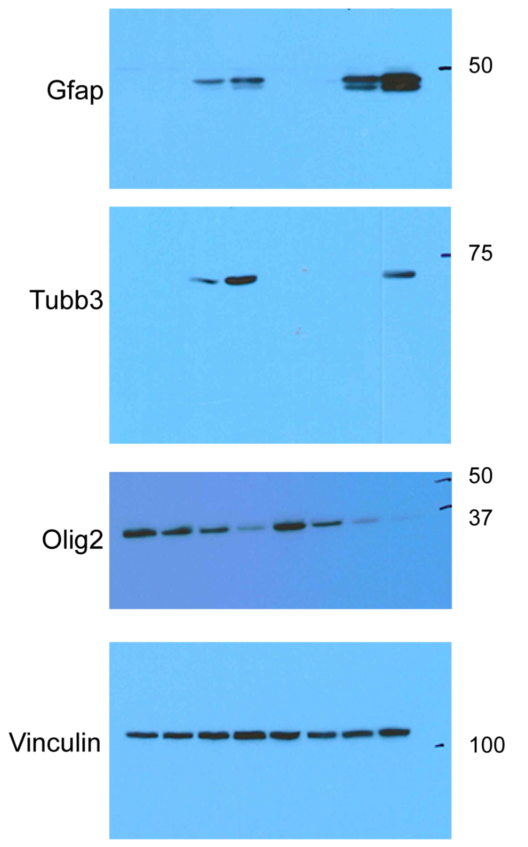

Figure 2c

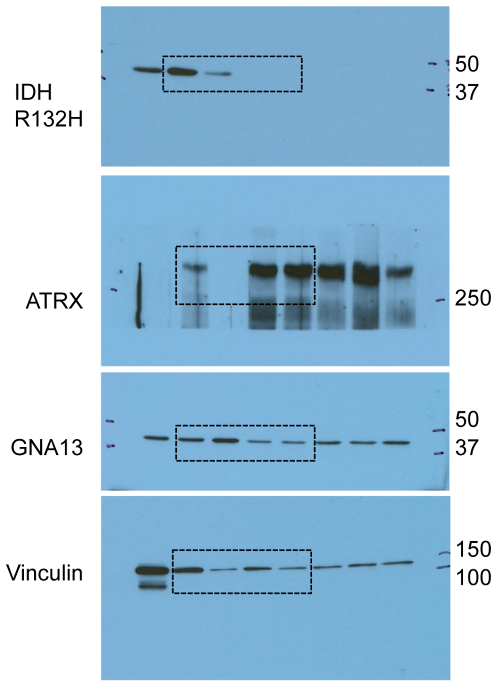

Figure 7a

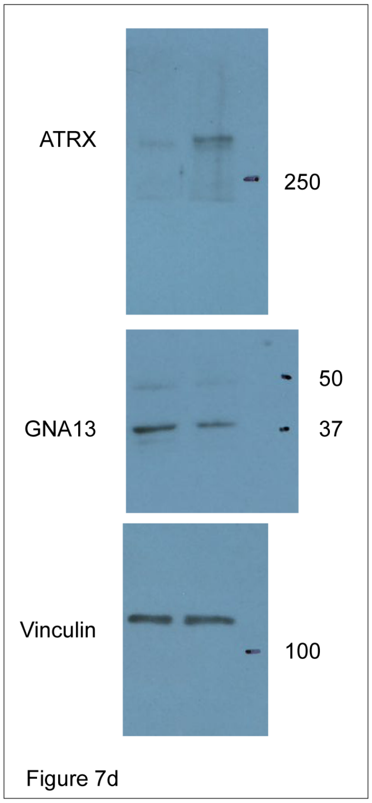

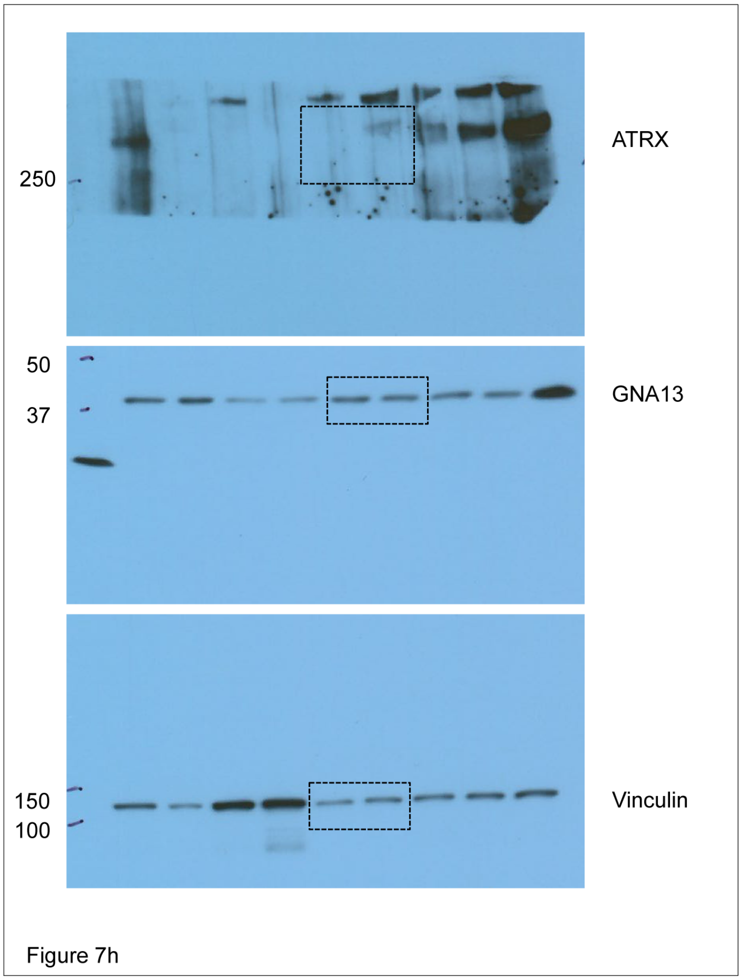

Supplementary FIG. 10: Uncropped western blots used in main paper figures. Molecular markers and paper figures associated with blots are indicated, as is the extent of horizontal cropping, when applicable. Molecular weight markers are in kD.
