# Supplementary material for: Atrx inactivation drives disease-defining phenotypes in glioma cells of origin through global epigenomic remodeling
Source: Nat Commun. 2018 Mar 13;9:1057. doi: 10.1038/s41467-018-03476-6 (PMC5849741; doi:10.1038/s41467-018-03476-6)
Supplement: Supplementary file 3 — Description of Additional Supplementary Files [file 41467_2018_3476_MOESM3_ESM.pdf]

Description of Additional Supplementary Files

File Name: Supplementary Data 1
Description: Differential gene expression and associated Atrx ChIP-seq peaks and ATAC-seq open and closed regions. Fold change between Atrx- and Atrx+ mNPCs is shown along with associated P value. Associations with Atrx ChIP-seq peaks and ATAC-seq open and closed regions (red) are determined by summing enrichment peak area within a ± 10 kb window surrounding the transcriptional start site in question. Transcriptional start and stop sites are also shown.

File Name: Supplementary Data 2
Description: Atrx ChIP-seq enrichment peaks for Tp53+/+ mNPCs.

File Name: Supplementary Data 3
Description: Atrx ChIP-seq enrichment peaks for Tp53-/- mNPCs.

File Name: Supplementary Data 4
Description: ATAC-seq open regions arising with Atrx deficiency in mNPCs.

File Name: Supplementary Data 5
Description: ATAC-seq closed regions arising with Atrx deficiency in mNPCs.

File Name: Supplementary Data 6
Description: Genomic sites of significant differential H3.3 composition arising with Atrx deficiency in mNPCs.

File Name: Supplementary Data 7
Description: Primers used for genotyping and RT-qPCR in this study.

File Name: Supplementary Data 8
Description: Primers used for ChIP-qPCR in this study
